# The structure of near-wall re-entrant flow and its influence on cloud cavitation instability

**DOI:** 10.1007/s00348-022-03417-6

**Published:** 2022-04-27

**Authors:** Udhav Gawandalkar, Christian Poelma

**Affiliations:** Multiphase Systems, Process & Energy (3mE), Delft University of Technology, Delft, The Netherlands

## Abstract

**Abstract:**

The so-called ‘re-entrant jet’ is fundamental to periodic cloud shedding in partial cavitation. However, the exact physical mechanism governing this phenomenon remains ambiguous. The complicated topology of the re-entrant flow renders whole-field, detailed measurement of the re-entrant flow cumbersome. Hence, most studies in the past have derived a physical understanding of this phenomenon from qualitative analyses of the re-entrant jet. Thus, quantitative studies are scarce in the literature. In this work, we present a methodology to experimentally measure the re-entrant flow below the vapour cavity in an axisymmetric venturi. The axisymmetry of the flow geometry is exploited to image tracer particles in the near-wall re-entrant flow. The main objective of employing tomographic imaging and subsequent velocimetry is to resolve the thickness and the velocity of the re-entrant flow. Additionally, phase-averaging conditioned on cavity length sheds light on the temporal evolution of re-entrant flow in a shedding cycle. The measured re-entrant film is as thick as $\sim 1.2$ mm for a maximum cavity length of $\sim 0.9 D_{t}$, where $D_{t}$ is the venturi throat diameter. However, the re-entrant film thickness at higher cavitation number is measured to be about 0.5 mm. Further, the re-entrant flow is seen to attain a maximum velocity up to half the throat velocity as the vapour cavity grows in time and the re-entrant flow thickens. We observe that a complex spatio-temporal evolution of re-entrant flow is involved in the cavity detachment and periodic cloud shedding. Finally, we apply the demonstrated methodology to study the evolution of the near-wall liquid flow, below the vapour cavity in different cavity shedding flow regimes. The role of two main mechanisms responsible for cloud shedding, i.e. (i) the adverse-pressure gradient driven re-entrant jet, and (ii) the bubbly shock wave emanating from the cloud collapse are quantitatively assessed. We observe that the thickness of the re-entrant liquid film with respect to the cavity thickness can influence the cavity shedding behaviour. Further, we show that both the mechanisms could be operating at a given flow condition, with one of them dominating to dictate the cloud shedding behaviour.

**Graphical abstract:**

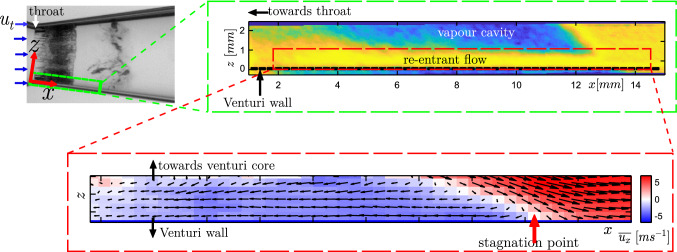

**Supplementary Information:**

The online version contains supplementary material available at 10.1007/s00348-022-03417-6.

## Introduction

Hydrodynamic cavitation is a phase change from liquid to vapour when the local pressure in the flow drops below the vapour pressure. Cavitation often occurs in turbomachinery and hydraulic equipment, such as ship propellers, pump impellers (Kuiper 1997), and even in diesel injectors (Giannadakis et al. 2008). Partial cavitation is a common form of cavitation that is characterised by unstable vapour cavities, which are intermittently shed. The larger vapour cavities are often shed periodically, resulting in cloud cavitation. This cloud cavitation is known to cause detrimental effects like erosion wear, material fatigue, noise, and vibration due to unsteady loads, which can all affect reliability and the lifetime of the equipment (Brennen 1995). On the other hand, cavitation also finds beneficial applications in the pre-treatment of biomass (Nakashima et al. 2016), water disinfection (Jyoti and Pandit 2004) and other chemical processes. Hence, understanding the fundamentals of cloud cavitation is imperative to manipulate its effects.

**Fig. 1:**
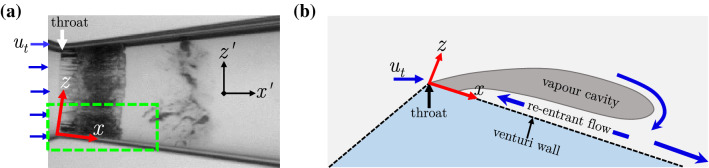
**a** A shadowgraph of a cavitating venturi, dark and light regions indicate vapour and liquid phase, respectively; **b** Schematic zoomed-in view of the vapour cavity and the re-entrant flow corresponding to the mid-plane in the green dashed box. The bulk flow is from left to right. Note the different coordinate systems: $x-z$ is aligned with the venturi wall, while $x'-z'$ is the laboratory reference frame

The ‘re-entrant jet’ travelling beneath the vapour cavity is crucial for understanding periodic cloud shedding (Knapp 1958). The topology of the re-entrant flow is such that it exists as a thin liquid film wedged between the solid boundary and vapour cavity (see Fig. 1). Classically, it is assumed to be periodically generated at the cavity closure region when the vapour cavity has assumed its maximum length in a shedding cycle (Furness and Hutton 1975). It then travels upstream, i.e. opposite to the bulk flow direction, with a velocity of similar magnitude as the bulk velocity until it triggers cavity detachment. This detached cloud travels downstream and collapses in the region where pressure has recovered. At the same time, a new vapour cavity starts growing. Several experimental and numerical studies have explored the role of the re-entrant jet in cloud cavitation in a variety of cavitating flows. The external flows include flow over wedges (Lush and Skipp 1986; Ganesh et al. 2016; Stutz and Reboud 1997; Laberteaux and Ceccio 2001b; Gnanaskandan and Mahesh 2016), hydrofoils (De Lange and De Bruin 1997; Saito et al. 2007; Kubota et al. 1989; Foeth et al. 2006; Dular et al. 2005; Pham et al. 1999; Kawanami et al. 1997), divergent steps (Callenaere et al. 2001; Trummler et al. 2020), while internal flows include 2D nozzles (Furness and Hutton 1975; Pelz et al. 2017), venturis (Gopalan and Katz 2000; Barre et al. 2009; Jahangir et al.^2018^), and orifices (Stanley et al. 2014). However, the exact physical mechanism responsible for the formation of a jet and its role in cloud cavitation instability remains unclear.

Several studies highlight the conditions necessary for the generation of the re-entrant jet and the cloud cavitation instability. Callenaere et al. (2001) systematically varied the adverse pressure gradient to establish that a pressure gradient at the cavity closure region is necessary for the cavitation instability to occur, i.e. transition from stable sheet cavity to periodic cloud cavitation. Franc (2001), Gopalan and Katz (2000), Laberteaux and Ceccio (2001b), and Sakoda et al. (2001) also asserted that the pressure gradient below the vapour cavity was a function of the cavity closure position, i.e. cavity length. Hence, the presence of a re-entrant jet was seen to be dependent on the cavity length. Further, Coutier-Delgosha et al. (2007) found that the delay between the inception of the re-entrant jet and cavity break off is nearly constant, suggesting that cloud detachment depends only on the jet velocity, without any influence of cloud collapse. Hence, the correlation between adverse pressure gradient and the re-entrant jet development was established. Furthermore, Le (1993), using dye injection and high-speed visualizations, observed that dye injected near the cavity closure made its way to the leading edge of the hydrofoil in a cyclic manner. Kawanami et al. (1997) also performed experiments with a hydrofoil where an obstacle was placed to block the re-entrant jet. It was observed that blocking the re-entrant jet stopped cloud shedding, resulting in a frothy vapour mixture. Thus, the periodic nature of the re-entrant jet and its role in periodic cloud shedding was hypothesized (Pham et al. 1999; De Lange and De Bruin 1997).

On the other hand, Leroux et al. (2004) performed wall pressure measurements on a hydrofoil and observed intense pressure pulse near the cavity closure region. This was attributed to the shockwave produced by the collapse of the previously shed cloud. Thus, it was speculated that cavity destabilization and shedding were perhaps due to the interaction of the re-entrant jet and the shock wave. Further, Stanley et al. (2014) proposed that the flow re-attachment in the cavity closure region provides a transient pulse of momentum that drives the re-entrant liquid flow. In addition, a pressure pulse created by cloud collapse creates a reverse flow of larger velocity. Both the effects combine to produce a periodic travelling wave of constant velocity, leading to cloud cavitation. This was corroborated by Large Eddy Simulations of Trummler et al. (2020), who also proposed that flow reversal is correlated to the pressure peaks observed in the downstream region of the step nozzle. Consequently, there is no consensus on the physical mechanism driving the re-entrant jet initiated cloud shedding.

It can be argued that the lack of consensus is due to the dearth of experimental data for near-wall liquid flow, below the vapour cavity. There have been previous attempts to measure the velocity of the re-entrant flow to understand the re-entrant flow driven cloud shedding. However, the flow geometry of re-entrant flow poses a major challenge in studying it experimentally and numerically. It occurs as a thin film close to the wall. Hence, most of the numerical models aimed at understanding global cavitation behaviour likely do not resolve it in sufficient detail (Brunhart et al. 2020). At the same time, whole-field laser-based optical measurement techniques such as particle image velocimetry (PIV), laser-induced fluorescence (LIF) are limited by strong reflections, occlusion of laser illumination and opacity due to the cavitation cloud (Poelma 2020). Moreover, optical access below the vapour cavity in canonical 2D geometries such as 2-D wedges and venturis, hydrofoil is limited. Single point measurement techniques have been employed in the past to measure the velocity and thickness of the re-entrant jet beneath the cavity. Callenaere et al. (2001) measured the re-entrant flow thickness at a fixed point using an ultrasound probe for different cavitation conditions and reported that the ratio of re-entrant flow thickness to vapour cavity thickness is an important parameter that governs the cavity shedding dynamics. Pham et al. (1999), using surface electrical impedance probes, measured the velocity of the re-entrant jet at various axial positions below the vapour cavity. They reported that the re-entrant flow velocity was of the same order as that of the free stream velocity. Further, Stutz and Reboud (1997) and Barre et al. (2009), using a double optical probe, reported a similar finding. They also asserted that the velocity of the jet was not constant.

Foeth et al. (2006) and Laberteaux and Ceccio (2001b) performed PIV measurements of the cavitating flow over a wedge and hydrofoil, respectively. However, near-wall velocity fields could not be measured beneath the vapour cavity and consequently in the re-entrant flow, due to strong reflections by the vapour cloud and the lack of optical access. Dular et al. (2005) also performed PIV-LIF measurements to study cavitation flow structures and found only a handful of vectors pointing in the reverse direction at the interface of the vapour cavity. The majority of studies rely on high-speed shadowgraphy to track bubbles (Stanley et al. 2014) in the re-entrant flow, or they track the cavity deformations by the re-entrant flow (chaotic interface) (Callenaere et al. 2001; Sakoda et al. 2001; Barbaca et al. 2019; Jahangir et al. 2018) to qualitatively infer the velocity of the re-entrant jet. Thus, direct and complete measurements of re-entrant flow have not been reported in the available literature.

In this contribution, we aim to provide quantitative information of re-entrant flow in a cavitation flow, to examine its role in periodic cloud shedding. Three modalities are used to unveil the re-entrant flow dynamics: high-speed shadowgraphy, tomographic imaging, and planar PIV. Shadowgraphy, being qualitative, is ideal to study large-scale phenomena such as cavity front growth and cloud shedding. However, the underlying physics, such as the re-entrant jet dynamics, is quantitatively studied using velocimetry. We show that the axisymmetry of the venturi can be used to achieve direct optical access below the vapour cavity.

The flow topology of the re-entrant flow is further exploited to implement tomographic imaging to evaluate the re-entrant flow thickness and velocity fields. This allows us to capture the spatio-temporal evolution of the re-entrant flow. Furthermore, it is demonstrated that the flow velocity of the thin liquid flow beneath the vapour cavity can also be reliably measured using planar PIV, if the flow thickness information is not needed. This not only helps in deepening our understanding of re-entrant jet initiated cloud shedding, but also provides acumen into the physics of other shedding behaviours such as re-entrant flow initiated aft cavity shedding and bubbly shock driven cloud cavitation occurring in a cavitating venturi. The velocity data generated can further be used to validate numerical models aimed at capturing cavitation dynamics.

## Experimental methodology

### Flow facility

**Fig. 2:**
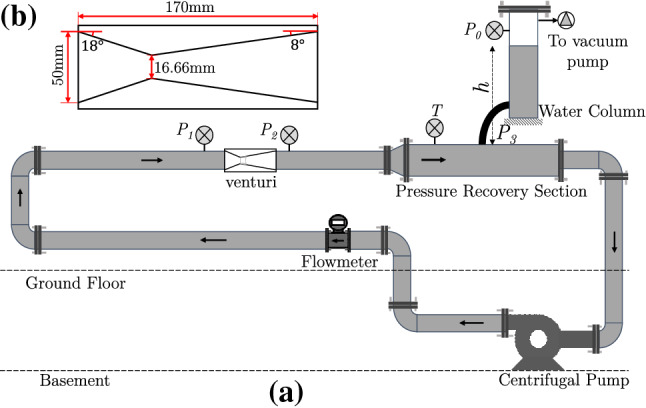
**a** A schematic of the cavitation loop in the Laboratory for Aero and Hydrodynamics, Delft. **b** The convergent-divergent axisymmetric venturi with geometric dimensions

The experiments are performed in the cavitation loop at the Laboratory for Aero and Hydrodynamics in Delft with water as a working fluid. The flow facility shown schematically in Fig. 2 has been described in detail in the previous work of Jahangir et al. (2018). Partial cavitation is realised at the throat of a venturi with a divergence angle of 8$^{\circ }$ and a throat diameter ($D_{t}$) of 16.67 mm. The cavitation behaviour in the venturi is governed by the cavitation number, $\sigma =(P_{3}-P_{v})/(\frac{1}{2}\rho U_t^{2})$, with $P_{v}$ the vapour pressure and $U_{t}$ the throat velocity. A lower $\sigma$ corresponds to more aggressive cavitation and vice versa. The vacuum pump in the flow loop allows independent control of the global static pressure ($P_{0}$) aside from the flow velocity ($U_{t}$), thus providing a wider range of $\sigma$. Further, the shedding frequency (*f*) is expressed as a Strouhal number, $St_{t}= fD_{t}/U_{t}$. A study of global cavitation behaviour revealed that re-entrant jet dominated cloud shedding occurs for: 0.95 $\le$ $\sigma$ < 1 (Jahangir et al. 2018). Hence, the experiments are performed for $\sigma \simeq$ 0.97, a Reynolds number defined at the venturi throat $Re_{t}\sim 170,000$, and $St_{t} \simeq$ 0.22 (shedding frequency of 133 Hz). The large-scale cavity shedding dynamics is visualised using high-speed shadowgraphy. The field of view (FOV) is centred along the venturi axis and spans 33 $\times$ 57 mm$^{2}$ in the $x'-z'$ plane. It is back-illuminated with a continuous white LED source. A high-speed CMOS camera (Photron Fastcam APX RS) equipped with an objective lens of 105 mm and aperture ($f^{\#}$) of 5.6 captures the contrast between the liquid (light) and vapour (dark) phase (see Fig. 1 a). The images are acquired at a rate of 12,000 Hz with an exposure time of 1/12,000 seconds.

### Imaging approach

**Fig. 3:**
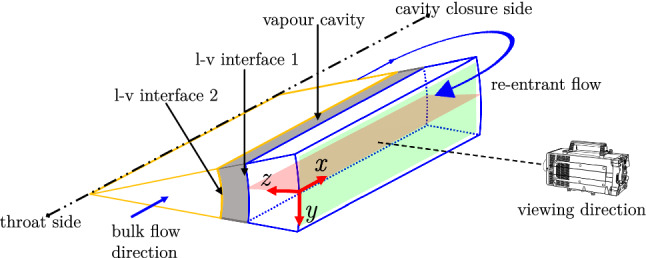
A schematic (not to scale) showing the measurement volume of the re-entrant liquid film, highlighted by the blue edges (the $x-z$ plane is shown in red). The grey volume is the vapour cavity, bound by the bulk flow (liquid) shown in yellow edges and the re-entrant jet. The respective liquid-vapour (l-v) interfaces are also indicated. The venturi wall is not shown, but bounds the re-entrant jet on the camera side

The topology of the re-entrant flow in a cavitating venturi makes its whole-field optical measurement challenging. Ideally, the re-entrant flow is measured in the mid-plane of the venturi (see Fig. 1a). However, the proximity of the flow close to the wall and strongly reflecting nature of the vapour cavity results in particle image contamination due to intense glaring and reflections (Laberteaux and Ceccio 2001a; Dular et al. 2005). Additionally, the opaque vapour cavity restricts optical access to the re-entrant flow (Foeth et al. 2006; Gopalan and Katz 2000). To circumvent these issues, the optical access to the re-entrant flow is achieved from the front side (see viewing direction in Fig. 3). This is facilitated by the axisymmetry of the venturi and time-averaged re-entrant flow attributes, such as the flow velocity and the thickness. Hence, a slender measurement volume is chosen, extending in the *x*-direction and relatively short in the *y* and *z*-direction. The extent of the *z*-direction is dictated by the re-entrant flow thickness while, the extent of *y*-direction is < 6$\%$ of the local circumference of the venturi. Thus, the choice of the FOV minimizes the effect of the curvature of the venturi and optimizes the image acquisition frequency to resolve essential flow dynamics.

Broadly, two imaging approaches are employed: Firstly, tomographic imaging of tracer particles is performed with multiple views followed by reconstruction of particles in the measurement volume (see volume indicated by blue boundaries in Fig. 3). We do not expect variation in the *y*-direction for the averaged behaviour, hence the *y*-direction is used as averaging direction. To achieve this, the reconstructed particle image intensities are projected on the $x-z$ plane. The velocity vector fields are also evaluated in this $x-z$ plane, shown in red. Thus, the re-entrant flow in front of the vapour cavity can be resolved. Secondly, in separate experiments, planar PIV is also performed on particle images in the $x-y$ plane. Here, the evaluated velocity fields are (inherently) averaged in *z*-direction, i.e. along the thickness of re-entrant flow.

**Fig. 4:**
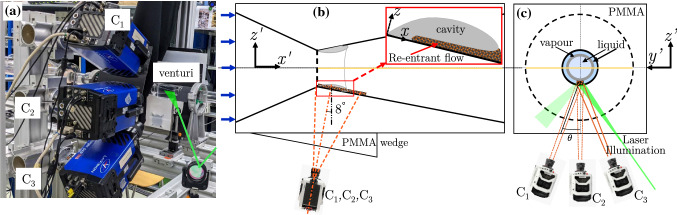
**a** Experimental setup for tomographic imaging. **b** Schematics of tomographic imaging setup in $x'-z'$ plane. The inset shows a zoomed-in view of re-entrant liquid film and vapour cavity in $x-z$ plane. The bulk flow is from left to right **c** $y'-z'$ plane shows the laser illumination

### Tomographic imaging

Tomographic imaging is performed with the objective to measure the thickness and the velocity of the re-entrant flow. The axisymmetry of vapour cavity and re-entrant flow is exploited to gain optical access to the re-entrant jet, as shown in Fig. 3, and Fig. 4b, c. Three high-speed cameras (LaVision Imager HS 4M) equipped with an objective lens of 105 mm ($f^{\#}= 5.6$) and high-pass optical filter ($\lambda>$590 nm) provide multiple imaging views. The spatial resolution of the image corresponds to approximately 0.038 mm per pixel. The cameras are arranged in a linear configuration ($\theta$ $\simeq$ $27^{\circ }$,$0 ^{\circ }$, $-27 ^{\circ }$) in the $y'-z'$ plane as shown in Fig. 4a, c. Such a configuration is chosen due to the elongated measurement volume. The cameras are mounted such that they make an angle of 8$^{\circ }$ in the $x'-z'$ plane to account for the divergence angle of venturi. This is complemented by a polymethylmethacrylate (PMMA) wedge of the same angle to reduce image distortion due to refraction at the outer surface of the venturi. Furthermore, scheimpflug adapters are used to align mid-planes of the illuminated area with the focal planes of the cameras.

The liquid flow is seeded with fluorescent tracer particles (‘FLUOSTAR’, acrylate resin particles coated with Rhodamine B, 13$\mu$m diameter) which absorb green light and emit orange light. The use of these orange fluorescent particles, in combination with a high-pass optical filter to block the green light of lower wavelength, eliminates the spurious reflections and glare arising from the vapour cavity. The volume illumination of particles is achieved by a Nd:YLF laser (25mJ per pulse at 1 kHz and 527 nm wavelength, Litron Lasers) introduced from the front side of the venturi and diverged using a plano-concave lens as shown in Fig. 4a,c. Interestingly, the re-entrant flow is enclosed by the venturi wall and the vapour cavity. Hence, the measurement volume is naturally formed by venturi wall on one side and vapour cavity on the other (see inset in Fig. 4b). Thus, knife-edge filters are not required. The effective measurement volume spans $\sim$ 24$\times$3.2$\times$2.8 mm$^{3}$. For each measurement 20,000 images are acquired, at a rate of 17.9kHz to achieve the required particle image displacement (< 6 pixels). Tap water is used as working fluid, which is expected to be saturated with cavitation nuclei. This implies that there are sufficient sites for cavitation to occur if the local hydrodynamics condition demand it, and adding (tracer) particles beyond this saturation level has no influence on the cavitation dynamics. To ensure this, $St_{t}$ as a function of $\sigma$ is examined before and after adding tracer particles at several global static pressures. The comparison showed good agreement (within the scatter of the data), hence the measurements are adjudged to be non-intrusive.

### Data processing

All data handling and processing is performed using DaVis 8.4 (LaVision GmbH). First, the acquired particle images are pre-processed to remove the background intensity by subtracting the temporal sliding minimum. Further, particle image intensities are normalised using min-max filtering (Westerweel 1997). Volume self-calibration (Wieneke 2008) is performed to reduce the calibration error to $\sim$ 0.03 pixel. This further aids in minimizing particle intensity reconstruction errors. The particle intensities are then reconstructed in the measurement volume using an iterative MART (multiplicative algebraic reconstruction technique) algorithm with six iterations and a relaxation parameter of 1. Further, the projections of the particle image intensity in the $x-z$ plane are computed to visualise the re-entrant flow. The global particle seeding density is maintained low ($\sim$ 0.03 g/L, ${\mathcal {O}}$(20) particles per mm$^3$) as to not influence global cavitation dynamics. Note that the mentioned seeding density is without accounting for the unavoidable settling of particles in the flow loop. Therefore, the effective particle density is increased by phase-averaging, conditioned on the cavity length in a shedding cycle (explained later in Sect. 2.6). If this averaging approach would not have been pursued, unfeasible levels^1^ of seeding would be required for the current magnification. Further, it is expected that the time-averaged velocity variation in the *y*-direction is negligible. Hence, the velocity fields in $x-z$ plane are evaluated from the particle image projections, with a multi-pass interrogation approach such that the final interrogation window size is 12$\times$12 pixels with 50$\%$ overlap. This is followed by spurious vectors elimination via universal outlier detection (Westerweel and Scarano 2005). Removed outliers are not replaced by interpolation, since only phase-averaged velocity fields are of interest. It is ensured that after vector elimination, each velocity vector is averaged over at least 90$\%$ of the samples at a given location.

### Planar particle image velocimetry

**Fig. 5:**
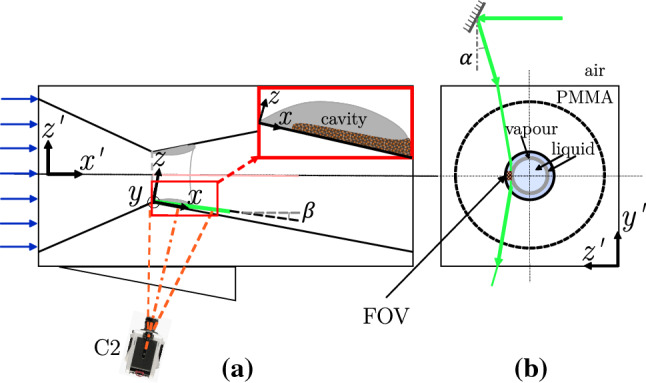
A schematic of the experimental setup for planar PIV: **a** in the $x'-z'$ plane, **b** in the $y'-z'$ plane, with light sheet path. The bulk flow is from left to the right

The planar PIV setup is identical to the one used for tomographic imaging, yet now using a single camera ($C_{2}$, see Fig. 5). In principle, the tomographic images could have been processed to obtain similar velocity data, by averaging in the *z*-direction (see Fig. 3). The main reasons for these additional planar PIV experiments is to verify if they can provide similar information in future studies, while being considerably easier to implement.

The flow conditions ($\sigma$ and $U_{t}$) are the same as for the tomographic measurements. The significant difference in the planar PIV measurement is the light sheet illumination. Exploiting the flow topology, i.e. the axisymmetry of the re-entrant flow and the thin nature of the liquid film, the light sheet is introduced in the re-entrant flow at an angle $\alpha$ with respect to the $y'$ axis. Further, the light sheet is oriented at an angle of $\beta$ with respect to the *x*-axis to account for the varying path-length in water due to the diverging geometry of venturi (see ray diagram in Fig. 5). Such an arrangement ensures that the light-sheet is refracted as shown in Fig. 5b, illuminating the liquid film enclosed by the vapour cloud and the venturi wall. The flow is seeded with the same fluorescent tracers as mentioned previously (Sect. 2.3). The camera is equipped with an objective lens of 200 mm ($f^{\#}=4$) and a high-pass orange optical filter. It is placed normal to the *x*-axis such that images are recorded in the $x-y$ plane. The FOV is centred at *y*-axis, spanning 22$\times$3.4 mm$^{2}$. A total of 15,000 images are acquired at 18,000 Hz with an exposure of 1/18,000 seconds. To evaluate the velocity vector fields, a multi-pass interrogation approach is followed with a final interrogation window size of 32$\times$32 pixels (50$\%$ overlap). The vector fields are post-processed by vector validation using universal outlier detection (Westerweel and Scarano 2005). It is possible that the thin laser sheet is scattered by the vapour cloud, illuminating the entire liquid film in *z*-direction. However, due to the small thickness of the re-entrant film and low velocity gradients in *z*-direction (shown later), the velocity fields in $x-y$ plane can be deemed accurate.

### Conditional phase averaging

**Fig. 6:**
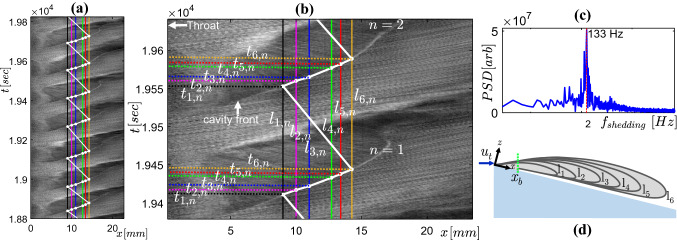
**a** A spacetime ($x'-t$) plot showing the cavity front growth. Only 7 shedding cycles are shown for clarity. **b** Detail of the $x'-t$ plot showing five different cavity lengths: 9 mm, 10 mm, 11 mm, 12.7 mm, 13.4 mm, and 14.3 mm, corresponding to $t/T = 0.3, 0.34, 0.38, 0.48, 0.54,$ and 0.58 over which data (tracer particle distribution and velocity) is phase-averaged. **c** Power spectral density showing periodicity in the cloud shedding process. **d** A schematic showing the cavity growth, $x_{b}$ corresponds to the length of streak-type bubble

The cloud shedding phenomenon is periodic in nature (see Fig. 6c for the dominant peak in the power spectral density (PSD) of the image intensity time series). Therefore, we study the dynamics of the re-entrant jet, and its role in periodic cloud shedding in a phase-averaged sense. The phase-averaging also helps in augmenting the signal-to-noise ratio of the data (velocity, projections) to discern the temporal evolution of re-entrant jet. We employ a conditional phase-averaging approach, where the velocity fields and particle image projections are averaged conditioned on the cavity length. This is justified since the attached cavity front grows with a constant velocity. To determine the phase, the spacetime ($x-t$) diagram of the vapour cavity front is constructed from the time-resolved raw PIV particle images (frontal view, C$_2$, see Fig. 4). Note that we choose a rectangular window centred about the *x* axis in each image. Further, we average the image intensity along the *y*-direction (see Fig. 3 for the coordinate system) such that for every time instance, we have the axial (*x*) extent of the vapour cavity. These are then stacked along a vertical time (*t*) axis, resulting in an $x-t$ plot shown in Fig. 6a. The bright cavity front can be discerned in the $x-t$ diagrams, indicating the length of the vapour cavity in a shedding cycle. Six cavity lengths in each cycle ($l_{1}...l_{6}$) are selected: 9 mm, 10 mm, 11 mm, 12.7 mm, 13.4 mm, and 14.3 mm, corresponding to $t/T = 0.3, 0.34, 0.38, 0.48, 0.54$, and 0.58 (see schematic in Fig. 6d). Here, *T* corresponds to the time period of the shedding process derived from the PSD (see Fig. 6c). Further, vertical lines corresponding to the above lengths ($l_{1}...l_{6}$) are plotted on the $x-t$ diagram and its time of occurrence is noted on the ordinate ($t_{1, n}...t_{6, n}$), where *n* corresponds to the number of the shedding cycle (see Fig. 6b). Further, two samples on either side of each ordinate ($t_{1, n}, ... t_{6, n}$) are included for averaging to augment signal-to-noise ratio. Finally, the velocity fields and projections for each cavity length ($l_{1}...l_{6}$) are averaged over 75 independent shedding cycles. This approach assumes that cycle to cycle variation is negligible in the cavity growth stage that is of interest ($t/T=0.3-0.58$, see Fig. 6). The cycle to cycle variation was quantified by the variation in the maximum cavity length and the cavity front growth rate per shedding cycle. The cavity front growth velocity in each cycle was estimated from the upward sloping cavity front in the $x-t$ plot, as shown in Fig. 6b. The vapour cavity growth rate was estimated to be 3 ± 0.22 ms$^{-1}$ (variation of < 7.5 $\%$), while the maximum vapour cavity length ($l_c$) had a variation of < 9$\%$. Further, variation in the phase-averaged axial velocity ($\overline{u_{x}}$) for multiple time instances is within 10$\%$. Moreover, a convergence study of the phase-averaged velocity shows that the relative change in velocity is less than 1.5 $\%$ (data not shown here) for the number of samples considered.

## Results

### Global shedding dynamics: a qualitative analysis

Firstly, we analyse the global shedding dynamics of re-entrant jet initiated cloud cavitation. This will allow us to identify a region of interest in a cavity shedding cycle to probe the re-entrant jet dynamics. Since the vapour cavity evolves both in space (*x*) and time (*t*), the $x-t$ diagram is an ideal tool to study large-scale shedding dynamics in a qualitative sense. Additionally, the inverse of the slope of a line in the $x-t$ diagram can be used to get a rough estimate of the cavity front growth rate.

**Fig. 7:**
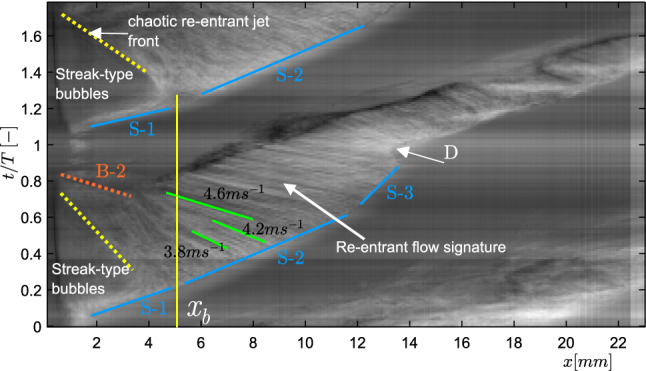
A spacetime ($x-t$) diagram of a single shedding cycle for $\sigma =0.97, U_t=13.56$ $ms^{-1}$. The bulk flow is from left to right

A single shedding cycle for the case of re-entrant jet-driven cloud cavitation ($\sigma =0.97$, $U_t=13.56$ $ms^{-1}$) is considered for in-depth analysis (see Fig. 7). It is observed that the vapour cavity typically grows in three stages.

S-1 corresponds to the growth of streak type bubbles near the throat, which are seen for $0 < t/T \le 0.2$ in a cycle (see yellow vertical line at $x=x_{b}$ in Fig. 7). Such attached bubbles have been widely reported in the literature in other cavitating flow geometries, such as Pelz et al. (2017). In the next stage, $0.2 < t/T \le 0.6$, the cavity grows at a nearly constant velocity, as evident from the linear cavity front in the S-2 stage (see the second blue line in Fig. 7). During this stage, re-entrant jets are seen developing from the cavity closure region, travelling upstream towards the attached bubbles. These are visualised by the green solid lines in Fig. 7, observed also by De Lange and De Bruin (1997) and Sakoda et al. (2001). Further, during the S-3 stage, the vapour cavity is seen to develop a discontinuity or a tear-up at about $x \sim x_{b}$ and the cavity growth rate is reduced. This is evident from the steeper cavity front shown by the blue line in the S-3 stage. Moreover, the vapour cavity breaks away from the streak bubbles and the remaining streak bubbles retract, as represented by B-2 (orange dotted line in Fig. 7). However, this retraction of streak bubbles is quite abrupt in some shedding cycles. Furthermore, *D* marks the end of bubble retraction, indicating the complete detachment of the cavity. Post this, the shed cloud is convected downstream with considerable swirl into the high-pressure region, where it eventually collapses. The latter event is outside the domain shown in Fig. 7. In the meantime, a new vapour cavity starts growing at the throat. This marks a complete shedding cycle for the current cavitation regime.

It is possible to estimate the velocity of the re-entrant flow from the $x-t$ diagrams: when re-entrant flow travels upstream, it imparts deformations on the cavity surface, giving rise to upstream travelling flow structures. These are visualised by sloping green lines in the S-2 and S-3 region (see Fig. 7) of cavity growth. The inverse of the slope of these lines roughly indicates the velocity of re-entrant flow, i.e. the steeper the streak, the lesser is the velocity. Typical values range from 3.8 to 4.6 ms$^{-1}$ in this cycle (see Fig. 7). Several studies in the past have relied on such approach to estimate re-entrant jet velocity (Stanley et al. 2014; Sakoda et al. 2001; Callenaere et al. 2001). However, this approach measures the deformation on the cavity interface rather than the actual re-entrant flow velocity.

**Fig. 8:**
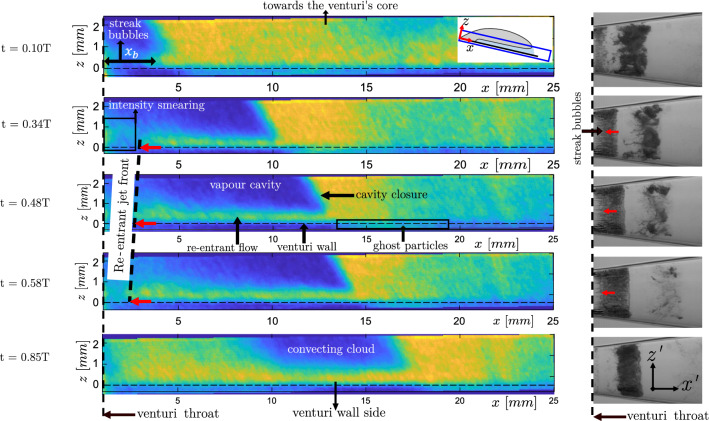
Left panel: phase-averaged re-entrant flow in the $x-z$ plane (colour indicates averaged concentration of tracer particle: dark blue patches indicates no particles in the vapour cavity, while green/yellow indicates liquid flow) for five different time instances ($t/T = 0.10, 0.34, 0.48, 0.58,$ and 0.85) in a shedding cycle. The horizontal black dashed line indicates the venturi wall. The inset in the top right corner indicates the FOV. Right panel: corresponding instantaneous shadowgraphs in the $x'-z'$ plane at the equivalent time instance in a shedding cycle. The red arrow marks the re-entrant jet front. See figure 1 for the coordinate systems. The bulk flow is from left to the right

### Re-entrant jet flow visualization

The temporal evolution of the re-entrant jet below the vapour cavity is visualized with phase-averaged particle image projections in the $x-z$ plane. See the inset in the left panel of Fig. 8 for the FOV and the coordinate system. Five time instances in a shedding cycle are considered, i.e. $t/T=0.1$, 0.34, 0.48, 0.58, and 0.85, (see left panel of Fig. 8). The colour in the contour plots indicates the phase-averaged intensity, $\langle I \rangle$. Yellow colours indicate high intensity, i.e. the presence of tracer particles. Conversely, blue indicates lower intensity, i.e. the absence of particles. By the virtue of the measurement technique and flow topology, tracer particles carried by the liquid phase are imaged in front of the vapour cavity. However, specular reflection on the cavity interface prevents the imaging of tracer particles inside the vapour cavity. Thus, tracer particles in the vapour cavity are not imaged and hence are not reconstructed. This results in two sharp interfaces; liquid-vapour (between re-entrant jet and vapour cavity) and liquid-solid (at the venturi wall). However, near the throat, smearing of $\langle I \rangle$ is observed. It is brought about by a different axial extent of the streak-type bubbles across different cycles (see Fig. 8). Note that the venturi wall is shown via a black dotted line. Further, no ghost intensities (Elsinga et al. 2006) are reconstructed in the vapour cavity. However, minimal ghost intensities can be seen outside the expected region, i.e. outside the venturi wall (see Fig. 8).

A comparison of the time series of re-entrant jet dynamics is made with the cavity front growth and shedding using instantaneous high-speed shadowgraphs at equivalent time instances (see right panel of Fig. 8). Note that these are not recorded simultaneously. The cavitation number is identical for both cases ($\sigma \sim$ 0.97). The dark and bright regions indicate vapour and liquid phase, respectively. Note that the coordinates systems are different in the left and the right panel, i.e. the orientation of the venturi in the left panel is such that it is rotated by an angle (8 $^{\circ }$) in an anticlockwise direction. The comparison shows that liquid-vapour phase separation is captured accurately throughout the shedding cycle, confirming the robustness of the phase-averaging methodology, despite minor intensity smearing due to cycle-to-cycle variation in cavity shedding. As the cavity front grows beyond the attached streak type bubbles ($x_{b}$), re-entrant liquid flow is seen to exist below the vapour cavity, in line with our shadowgraphy observations. During this time ($t/T = 0.1-0.58$), the re-entrant flow front travels upstream towards the throat, as shown in Fig. 8. Thus, the re-entrant jet is not periodically generated, rather it seems to be continually present below the vapour cavity for the most part of the shedding cycle. This is in contradiction to the classical description of the re-entrant flow, which suggests that re-entrant flow is periodically generated when the vapour cavity has assumed the maximum length (Knapp 1958). Finally, the re-entrant jet front can be seen in the phase-averaged flow visualizations (see black dotted line in the left panel of Fig. 8) moving upstream with velocity $\sim 0.12U_{t}$. This chaotic front is also seen in the high-speed shadowgraph (although less clearly). Similar observations were reported by Jahangir et al. (2018), Barbaca et al. (2019), and De Lange and De Bruin (1997), who interpreted it as the re-entrant jet velocity.

**Fig. 9:**
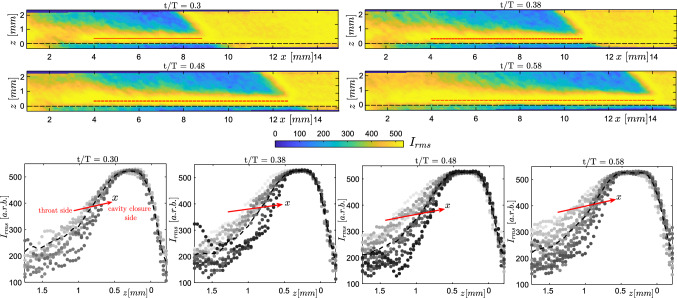
Re-entrant flow thickness evolution at $t/T=0.3$, 0.38, 0.48, 0.58. The black dotted line indicates the venturi wall. The bottom panel shows $I_{rms}$ profiles in the *z*-direction perpendicular to the red line in the axial direction (*x*)

### Re-entrant flow thickness

Callenaere et al. (2001) reported that the thickness of re-entrant flow with respect to the vapour cavity thickness is an important parameter that governs the cavity shedding dynamics. Hence, one of the major objectives of performing tomographic imaging of re-entrant flow was to quantify its thickness and its spatio-temporal evolution. This requires accurate identification of two interfaces that enclose the re-entrant flow: (i) the liquid-vapour interface at the free cavity surface, and (ii) the liquid-solid interface at the venturi wall. For this, we utilize the standard deviation in the particle image intensity, $I_{rms}$, akin to Reuther and Kahler (2018), who used a similar approach to identify the turbulent-non turbulent interface in a turbulent mixing layer. The standard deviation at a given time instance ($t/T=0.3$-0.58) is computed with respect to the phase-average intensity field $\langle I \rangle$ for that given phase.

The absence of tracer particles in the vapour region and outside the venturi results in a lower standard deviation in these regions (see Fig. 9). Conversely, tracer particles in the re-entrant film result in significant $I_{rms}$. This results in a large step change in the standard deviation of the intensity values across the liquid film. This approach allows us to resolve the re-entrant film with a higher resolution, i.e. $\sim$ ${\mathcal {O}}$(particle image size), rather than the velocity vector spacing, which is larger. It is imperative to consider the particle image size in the reconstruction, as it forms the basis of the re-entrant jet film thickness estimation. It is observed that due to the shallow viewing angles of the cameras (see Fig. 4c), dictated by the thin nature of the re-entrant flow, the particle intensities in the depth direction (*z*) are reconstructed at an angle and are slightly elongated ($\sim$ 7–8 pixels). This can be explained by an increased in-plane uncertainty in the 3D particle triangulation ($\delta$) defined as $\delta =d_{\tau }/$tan$(\theta /2)$, where $d_{\tau }$ and $\theta$ are the particle image diameter and angle between the optical axes of the cameras, respectively (Kim et al. 2013). For instance, for a particle image diameter of 2 pixels, $\delta$ could be almost 8 pixels. However, the estimated thickness of the re-entrant flow spans over 35-50 pixels. In our estimates, re-entrant flow thickness can be overestimated by $\sim$ 4-5 pixels. Closer to the throat ($x < 4$ mm), the $I_{rms}$ values are inflated by the aforementioned cycle-to-cycle variation in the phase averages, and hence the thickness data are deemed unreliable in this region (see top panel of Fig. 9). $I_{rms}$ profiles in the *z* direction are plotted perpendicular to the red dashed line in Fig. 9, such that re-entrant flow thickness as a function of axial distance (*x*) can be studied. The profiles with a lighter shade indicates *x* positions closer to the throat, while darker shades are further away from the throat, respectively.

**Fig. 10:**
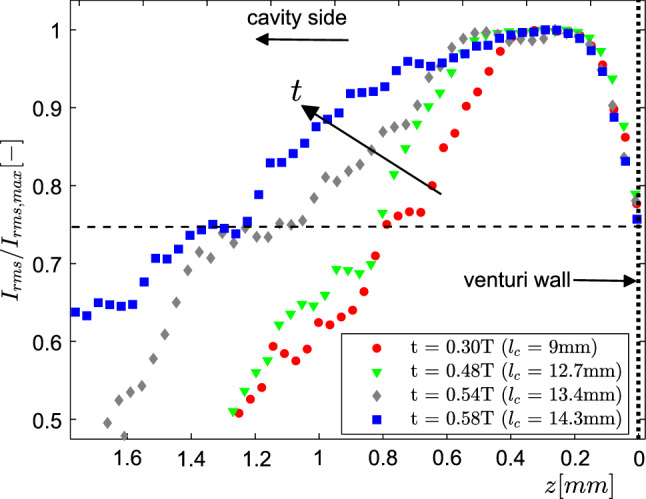
RMS of phase averaged particle image intensity profiles showing re-entrant flow thickness at different time-instances in a shedding cycle at a fixed axial location ($x/D_{t} = 0.36$). The $I_{rms}$ profile is normalised with the maximum value for comparison. The black dashed line shows the chosen threshold value of 0.75, while the black dotted line shows the position of the venturi wall

It is observed that re-entrant flow gets thicker as it progresses upstream, further away from the cavity closure region. This is evident from the $I_{rms}$ profiles, which get wider (compare the profiles in lighter shades with the darker shades in the bottom panel of Fig. 9). Moreover, as the cavity grows longer in time, re-entrant flow beneath it gets thicker, as clear from the flatter and wider averaged intensity profiles (see the lower panel of Fig. 9). This is further illustrated by $I_{rms}$ profiles at a fixed axial location ($x/D_{t}$ = 0.36) for different time instances (see Fig. 10). The RMS intensity is normalised with the maximum of the profile, while a threshold of 0.75 is chosen to quantify the thickness. For the considered time instances in a cycle, the re-entrant film thickness is seen to increase from 0.9 mm to 1.2 mm. The maximum re-entrant flow thickness in a cycle is estimated to be approximately 1.2 mm for a maximum cavity length of 0.9$D_{t}$. At this cavity length, the maximum cavity thickness is estimated to be 0.27$D_{t}$ ($\sim$ 4.5 mm) based on the X-ray measurements of Jahangir et al. (2019), for similar flow conditions. Thus, the re-entrant film thickness is estimated to be 26$\%$ of the vapour cavity thickness. This is in line with the ultrasound measurement of Callenaere et al. (2001), who reported this value to be in the range of 15$\%$ to 35$\%$. The described spatio-temporal variation of re-entrant flow thickness suggests that as the cavity grows in time and re-entrant flow travels further upstream near the throat, the re-entrant flow gets thick enough with respect to the vapour cavity thickness. This allows interaction of re-entrant flow with the vapour cavity (Callenaere et al. 2001), initiating the cavity pinch-off at $x \sim x_{b}$. This agrees well with the discontinuity observed in the vapour cavity at a similar axial position ($x \sim x_{b}$) in the S-3 stage of the shedding cycle (see Fig. 7).

**Fig. 11:**
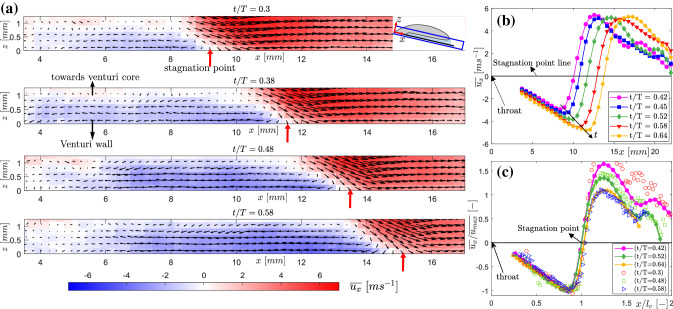
**a** Phase-averaged re-entrant flow velocity fields at $t/T=0.3$, 0.38, 0.48, 0.58, **b** Phase-averaged axial velocity variation evaluated with planar PIV along the *x*-direction during cavity front growth ($t/T=0.42$, 0.45, 0.52, 0.58, 0.63), **c** Comparison of normalised depth-averaged axial velocity variation with planar PIV at various time instances

### Re-entrant flow velocity

The liquid-vapour phase determination methodology described in the previous subsection does not make distinction between a stagnant liquid film and a re-entrant jet below the vapour cavity. Hence, quantifying the flow velocity corresponding to the re-entrant flow thickness is imperative. The velocity evaluation method from raw particle images has been explained in detail in Sect. 2.4, while the phase-averaging methodology is detailed in 2.6. The phase-averaged velocity field of re-entrant flow ($\overline{u_{x}}$) in the $x-z$ plane in the laboratory frame of reference at several time instances in a cycle ($t/T=0.3$, 0.38, 0.48, 0.58) are shown in Fig. 11. The bulk flow (‘red’) is from left to right, while the re-entrant flow (‘blue’ false colours) is from right to left. The velocity field shows that re-entrant flow is a consequence of an impinging jet and a stagnation point formed at the cavity closure region (see red arrows in Fig. 11a). It is seen that as the vapour cavity grows beyond the streak bubbles, the flow encounters a stagnation point near the cavity closure region and the re-entrant flow is swept below the vapour cavity. The stagnation point moves further away as the vapour cavity grows in time ($t/T=0.3$-0.58). Supplementary movie S1 shows a visualisation based on all reconstructed phases. During this time, the re-entrant flow is continuously fed by the bulk flow below the cavity, while the fluid on the other side of the stagnation point moves away from the throat. This is evident from the change of velocity direction past the stagnation point in Fig. 11a and b. The spatial variation of the re-entrant jet velocity shows that as the jet begins from the stagnation point, it starts accelerating towards the throat to achieve a maximum velocity ($\overline{u_{max}}$). However, at about $x/l_{c} \sim 0.85$, it starts slowing down (see Fig. 11c). Here, $l_{c}$ indicates the vapour cavity length. This is because the re-entrant jet is blocked by the streak type attached bubbles, as seen from the raw particle images and high-speed shadowgraphs (see Fig. 5). Such a decrease of velocity in the axial direction has also been reported by Pham et al. (1999), Sakoda et al. (2001) in other cavitation flows. As the vapour cavity grows in time, a jet with higher velocity is generated from the cavity closure point further away from the throat, as evident from Fig. 11. This is in-line with the earlier observations of Gopalan and Katz (2000), Laberteaux and Ceccio (2001b), Franc (2001), suggesting that re-entrant flow is adverse pressure gradient driven.

We observe that the maximum re-entrant flow velocity increases substantially from 2.5 ms$^{-1}$ to 5 ms$^{-1}$ for the throat velocity of 10.2 ms$^{-1}$ (see Fig. 11b). This is consistent with our velocity estimates from the $x-t$ plots (see Fig. 7). This is also in close agreement with Callenaere et al. (2001), Pham et al. (1999), who reported re-entrant jet velocity to be about half of the mean flow velocity ($U_{\infty }$) for a diverging step and hydrofoil, respectively. The width (*z*-direction) of reverse flow region indicates that the re-entrant flow gets thicker in time, as also stated in the earlier subsection. Moreover, the momentum of the re-entrant flow approaching the throat increases monotonically in time until the vapour cavity develops a discontinuity (also see Fig. 8). This corroborates the hypothesis that as the cavity grows in time, the re-entrant flow gets thicker. At the same time, a stronger re-entrant flow is pushed below the cavity. The combination of these effects is involved in causing a cavity pinching off and discontinuity in the vapour at $x \sim x_{b}$. Consequently, this leads to cloud shedding. We report that the maximum velocity of re-entrant flow is less than the bulk velocity ($U_{t}$). This is in contrast to potential flow theory, which predicts the re-entrant flow velocity to be *higher* than the bulk velocity ($u_{t}$) : $u_{jet} = U_{t}(\sqrt{1 + \sigma })$ (Furness and Hutton 1975). Interestingly, the averaged velocity ($\overline{u_{x}}$) normalized with the peak velocity ($\overline{u_{max}}$) and axial distance (*x*) normalized with the cavity length ($l_{c}$), collapse on top of each other, showing self-similarity for the re-entrant jet beneath attached cavities (see figure 11c).

### Data validation

While the re-entrant jet velocity and its spatio-temporal variation is resolved accurately in the axial direction (*x*) (see Fig. 11 b), its spatial resolution in the depth (*z*) direction is limited to 4–5 vectors. This is mostly brought about by the thin film topology of the re-entrant flow. The measurement technique is also limited by: (i) the diffraction-limited particle images wherein particle images appear bigger than their actual size, (ii) the finite resolution of PIV dictated by the interrogation window size. Furthermore, there is a limitation on tracer particle concentration to preserve non-intrusiveness of the measurements, since a large concentration of impurities (such as tracer particles) can alter the cavitation dynamics and also induce opacity to the flow.

**Fig. 12:**
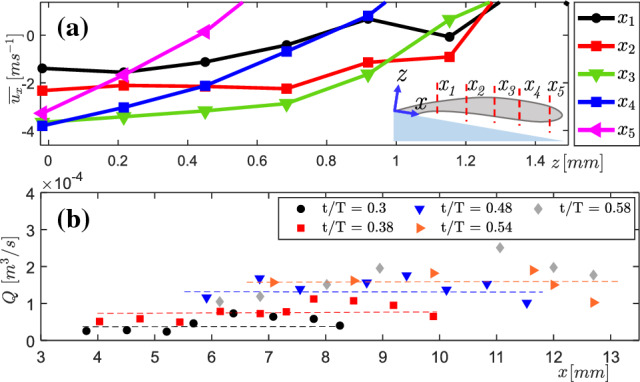
**a** Axial velocity profile development of the re-entrant flow at $t/T=0.48$ at various axial positions. **b** Volume flux variation of re-entrant flow along the axial direction for $t/T=0.3$-0.58

In order to assess the quality of the measured velocity data, the development of the axial velocity ($\overline{u_{x}}$) profile along the *x* direction is considered. A qualitative comparison of re-entrant flow is made with a submerged impinging jet flow (Fitzgerald and Garimella 1997), as the re-entrant jet flow field resembles it closely (see Fig. 11). The phase-averaged axial velocity profile ($\overline{u_{x}}$) at one time instance, $t/T=0.48$ ($l_{c} =12.7$mm), is treated for the sake of clarity at various axial positions (see Fig. 12a and the inset).

It is seen that the gradient of the velocity profile is higher near the cavity closure region, yet the profiles progressively get flatter as the jet approaches the throat due to the deceleration it experiences. Such evolution of the axial velocity profile agrees well with the observations of Fitzgerald and Garimella (1997). Further, due to the limited spatial resolution of PIV, the thin high-shear region close to the venturi wall could not be resolved (see velocity profile in Fig. 12). Assuming that re-entrant flow is axisymmetric in a phase averaged sense, the velocity profiles in the *z*-direction can be integrated along its thickness over the entire circumference of the venturi. This is done to verify mass conservation, i.e. the volumetric flow rate in the jet (*Q*) is constant for various x-positions (see Eq. 1). Here, $R_{i}(x)$ and $R_{o}(x)$ are the inner and outer radii of the annulus, formed by the re-entrant flow, which are both also a function of the axial position due to the diverging geometry of the venturi.

1 $$\begin{aligned} Q(x) = \int _{R_{i}(x)}^{R_{o}(x)} 2\pi \overline{u}(r, x) r (r,x) \;\mathrm {d}r \end{aligned}$$

The volume flux of the re-entrant flow at different axial positions at various time instances ($t/T = 0.3, ..., 0.58$) in a shedding cycle are shown in Fig. 12b. It is observed that at every time-instance, the re-entrant flow flux remains nearly constant (see Fig. 12b) except for *t*/*T* = 0.58, where cavity detachment is suspected to have an influence. This also confirms that the loss of velocity-data in the high-shear region is perhaps small. Further, as expected, the volume flux of the re-entrant jet increases monotonically with time as a stronger re-entrant flow is fed by the bulk flow (see Fig. 11a).

### Comparison of tomographic imaging and planar PIV

The planar PIV inherently yields depth (*z*)-averaged velocity fields in the $x-y$ plane, due to the finite light sheet thickness. Further, the velocity fields are phase-averaged conditioned on cavity length. The variation of the axial velocity component ($\overline{u_{x}}$) in the *y*-direction appears to be negligible. Hence, the vector field is further averaged in the *y*-direction to get the axial (*x*) variation of $\overline{u_{x}}$, as shown in Fig. 11b. In an independent experiment, the velocity fields are computed from *y*-averaged tomographic particle image projections, in the $x-z$ plane. These are also averaged along the re-entrant flow thickness (*z*-direction) such that the axial (*x*) variation of $\overline{u_{x}}$ is obtained.

When non-dimensionalised with the corresponding $\overline{u_{max}}$, and $l_{c}$, the one-dimensional variation of $\overline{u_{x}}$ shows a good agreement (see again Fig. 11c). Here, the $\overline{u_{x}}$ evaluated from planar PIV is shown by filled symbols while, the $\overline{u_{x}}$ evaluated from tomographic image projections are shown by open symbols. The maximum velocities at a given time instance are also comparable. Thus, it is observed that the velocity variation of the re-entrant flow is small along its thickness. This is also confirmed by the velocity profiles in Fig. 12. Hence, the re-entrant flow velocity beneath attached vapour cavities is seen to have a unidirectional variation. Consequently, planar PIV is expected to provide robust estimates of axial velocity variation in an axial direction.

## Application of the technique and discussion

A thorough understanding of cavitation physics is strongly driven by quantification of flow characteristics, such as the velocity field, pressure, and void fractions. Hence, the discussed methodology can now be applied to evaluate the velocity field of re-entrant flow below the vapour cavity in different cavity shedding flow regimes that exist in the axisymmetric venturi.

**Fig. 13:**
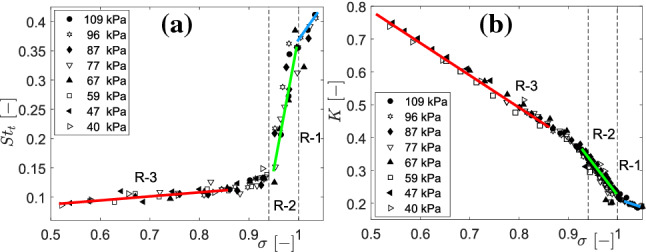
Cavitation flow regimes (R1, R2, R3): **a** Strouhal number ($St_{t}$), **b** Pressure loss coefficient (*K*) as a function of cavitation number ($\sigma$) at different global static pressures

Firstly, the global cavitation behaviour is studied by systematically varying the $\sigma$ to identify various flow regimes. The vapour cavity dynamics is characterized by the vapour shedding frequency (*f*) expressed as Strouhal number ($St_{t} ={fD_{t}}/{U_{t}}$) and the pressure drop across the venturi expressed as pressure loss coefficient, $K=({p_{1}-p_{2})/(\frac{1}{2} \rho U_{t}^{2}})$, see Fig. 2 for definitions. $St_{t}$ and *K* are plotted for various $\sigma$ at different global static pressures in the flow loop as shown in Fig. 13.

It is seen that the variation of the Strouhal number with $\sigma$ exhibits a change in slope at $\sigma \sim$ 1 and 0.95 (see Fig. 13a). This change in slope is well reproduced in the variation of the pressure loss coefficient as a function of $\sigma$ (see also Fig. 13b). This forms the basis of three distinct cavity shedding behaviour that will be discussed. As the intensity of cavitation is increased gradually, i.e. $\sigma$ is decreased, the following shedding behaviours are observed: (i) re-entrant flow initiated aft cavity shedding (R1: $\sigma$ $\ge$ 1), (ii) re-entrant jet initiated periodic cloud shedding (R2: 0.95 $\le \sigma < 1$), and (iii) bubbly shock driven periodic cloud shedding (R3: $\sigma \le$ 0.85) (see again Fig. 13). The R2 and R3 flow regimes have been discussed qualitatively by Jahangir et al. (2018). However, R1 has not been explored in the cavitating axisymmetric venturi, mainly because of its less severe effects when compared to periodic cloud cavitation.

**Fig. 14:**
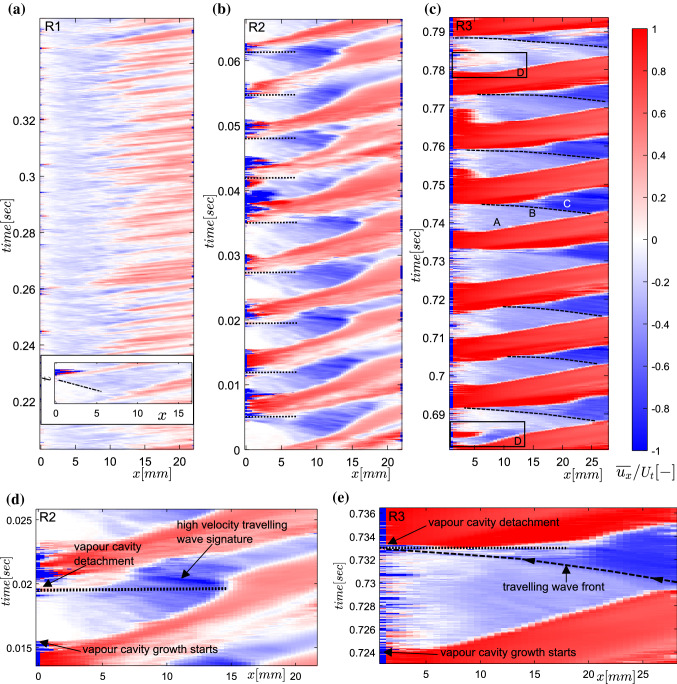
Flow regimes of partial cavitation in an axisymmetric venturi shown by spacetime plots **a** R1: re-entrant jet initiated aft cavity shedding ($\sigma =1.03$, $U_{t}=10.2ms^{-1}$) **b** R2: re-entrant jet initiated cloud shedding ($\sigma$ = 0.97, $U_{t}=10.4ms^{-1}$), **c** R3: Bubbly shock driven cloud shedding ($\sigma =0.82$, $U_{t}=9.9ms^{-1}$). (Black dashed profile indicates the front of bubbly shock wave), **d** zoom-in of a single shedding cycle in R2, **e** zoom-in of a single shedding cycle in R3. The bulk flow is from left to right

**Table 1:** Flow parameters for the studied flow cases

Flow regime	$P_{3}$ [kPa]	$U_{t}$ [ms$^{-1}$]	$\sigma [-]$
R1	57	10.2	1.03
R2	55	10.4	0.97
R3	43	9.9	0.82

A typical case of each flow regime is now examined with the velocity fields below the vapour cavity. The global static pressure in the system is varied while maintaining comparable bulk flow velocity at the throat in all three flow cases. As a result, three different cavitation numbers, covering the three regimes, are achieved (see Table 1). Due to the ease of implementation and demonstrated robustness, planar PIV is used. The velocity vector fields below the vapour cavity are evaluated in the $x-y$ plane, as discussed in Sect. 2.5. The axial velocity ($u_{x}$) is averaged along the *y*-direction to yield $\overline{u_{x}}$ as a function of *x*. The axial variation of $\overline{u_{x}}$ is then stacked on a time-axis to generate an $x-t$ evolution of axial velocity. These $x-t$ diagrams resemble those commonly used in cavitation research but are now encoded with the local velocity. Despite averaging of the velocity (${u_{x}}$) in the *y*-direction, the instantaneous flow structures are well-preserved in the spacetime evolution of $\overline{u_{x}}$. A few representative shedding cycles for each flow regime are shown in Fig. 14 for clarity. Here, red and blue indicates bulk (positive) and re-entrant flow velocity (negative), normalised with the throat velocity ($U_{t}$).

At the highest cavitation number considered, the vapour cavities are the thinnest and the shortest, as also reported by (Ganesh et al. 2016). We observe that the vapour cavity grows, rolls up, and gets fragmented into multiple smaller vapour cavities (R1, Fig. 14a). The cavity appears to be continually attached to the throat for spatial extent (*x*) < 2 mm. For $x > 2$mm, there is a continuous presence of re-entrant liquid flow below the cavity. This is evident from the patchy reverse flow structures in the spacetime plot of velocity. These reverse flow structures can extend longer in the axial direction in some shedding cycles (see long blue patches). This is because the reverse flow further away from the throat appears to be induced by the fragmented, smaller vapour structures. They carry significant swirl and consequently low pressure in their cores. Thus, they can sustain for a longer time, i.e. their collapse is delayed as they approach the high-pressure region of the venturi. The time-averaged thickness of re-entrant flow film in R1 is estimated to be about 0.5 mm from the tomographic imaging. Since the vapour cavities are thin with respect to the re-entrant flow thickness, the re-entrant flow is suspected to interact with the vapour cavity strongly. This interaction is involved in the fragmentation of the vapour cavity at multiple points along the axial direction, giving rise to the observed patchy flow structures. Thus, the vapour cavity is destabilised by the re-entrant flow, while re-entrant flow may not reach all the way upstream up to the throat. Consequently, it appears that the cavity is being shed from its aft side (closure region of the cavity) for the majority of instances. However, it also appears that the cavity is shed from the throat in some shedding cycles. This can be seen from the re-entrant jet front approaching the throat (see black dash-dotted profile in the inset of Fig. 14 a). This intermittency is responsible for disturbing the periodicity in the cavity shedding process. Such behaviour is also evident from a weaker peak in the power spectral density of the axial velocity time series (not shown here). Thus, vapour cavities appear to undergo shedding in a quasi-periodic manner. Such ‘quasi-stable sheet cavities’ or ‘oscillating thin cavities’ have been mentioned in the past by Callenaere et al. (2001) and De Lange and De Bruin (1997) on a diverging step and hydrofoil, respectively. More recently, Barbaca et al. (2019) reported such flow regime using X-ray densitometry over a wall-mounted fence. It was reported by Gopalan and Katz (2000), Leroux et al. (2004) that no re-entrant flow exists beneath the vapour cavity in this flow regime, besides a weak re-entrant flow at the cavity closure region. However, instantaneous velocity fields show that a reverse flow does exist ($\overline{u_{max}}\sim -0.25U_{t}$) below the majority of the length of the vapour cavity. Further, it is responsible for the shedding of the vapour cavity at multiple points along the cavity length.

As $\sigma$ is decreased (0.95 $\le$ $\sigma$ < 1), vapour cavities get longer and thicker. As the shedding cycle begins, the re-entrant liquid film is thin with respect to the vapour cavity. However, it evolves into a thicker film (as thick as $\sim$ 1.2 mm) as the vapour cavity grows in time. Hence, it can be argued that the interaction between the re-entrant flow and vapour cavity is delayed until the re-entrant flow is thick enough with respect to the vapour cavity. Consequently, a coherent re-entrant liquid flow is seen to exist below the vapour cavity for the majority of the cycle, in comparison to the more patchy reverse flow structures in R1. This can be seen from the $x-t$ diagram in Fig. 14b and flow visualizations in Fig. 8. The axial velocity variation in the $x-t$ diagram for R2 shows that as the vapour cavity grows, a stronger re-entrant flow is pushed below the vapour cavity. This is then followed by the cavity detachment. The cloud detachment instance for each cycle is shown by a black dotted line in Fig. 14b. The detached cloud then convects downstream with a significant swirl velocity. A maximum reverse velocity in the laboratory frame of reference ($\sim - 0.90 U_{t}$) below the vapour cavity is occasionally observed after the cavity has detached. This can possibly be explained by the travelling wave generated by the collapse of the cavitation cloud, which then gets superimposed on the existing re-entrant flow. The signature of this wave can be seen in Fig. 14d: the dark blue (high velocity) structure travelling upstream. However, it appears that cavity detachment triggered by the interaction of the re-entrant flow and the vapour cavity *precedes* the arrival of the travelling wave at the throat, as illustrated by Fig. 14d. In the current study, sharp pressure peaks were picked up by a high-speed dynamics pressure probe in the cavity collapse region (not shown here). This corroborates the presence of bubbly shock waves. In summary, we report the presence of high velocity upstream travelling waves of nearly constant velocity in the re-entrant jet dominated cavity shedding regime (R2). However, it is not a necessary condition for cavity detachment. Consequently, adverse pressure gradient driven re-entrant jet dynamics is here identified as a sufficient condition for the cavity detachment.

Further reduction of $\sigma$ leads to the largest vapour cavities (Jahangir et al. 2018). This results in a prominent bubbly shock wave emanating from the collapse of the large cavitation cloud. The signature of bubbly shock waves are registered by high-speed dynamic pressure sensor in the downstream region of the venturi. The distinct and high-pressure peaks are recorded periodically with a frequency identical to the cloud shedding frequency. These are further seen to trigger the cavity detachment and dictate the periodic cloud cavitation, as shown in Fig. 14c, Fig. 14e. For the majority of shedding cycles, the bubbly shock wave gives rise to an upstream travelling wave-like flow structure. This seems to be superimposed on the existing adverse pressure gradient driven re-entrant flow. A similar observation has also been reported by Stanley et al. (2014) as a phenomenological description of re-entrant jet initiated cloud shedding in a cylindrical orifice. However, the velocimetry confirms that this is an alternate mechanism for cloud cavitation instability, inline with Ganesh et al. (2016), Budich et al. (2018), and Jahangir et al. (2018). The wave front is visualized by a sharp jump in the axial velocity magnitude in the $x-t$ plot (see the black dashed curved profiles in Fig. 14c, e.g. near label B). It demarcates low velocity re-entrant flow region (A in Fig. 14c) and high velocity region (C).

**Fig. 15:**
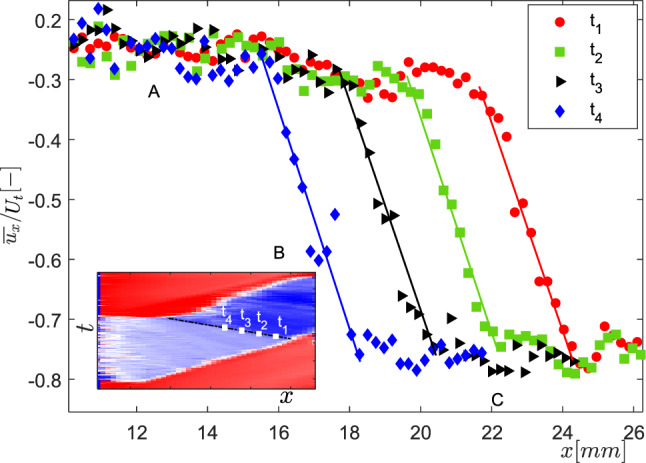
The instantaneous variation of $\overline{u_{x}}$ along the axial direction to show the propagation of velocity discontinuity towards the throat in time. A: reverse flow region due to the re-entrant flow, B: shock front, C: bubbly shock wave velocity

This is further illustrated by an $x-t$ diagram for a single shedding cycle in Fig. 14e. The dashed profile indicating the shock front travels upstream until the vapour cavity detachment point shown by a black dotted line. Further, the shock front is seen to travel at a near-constant velocity, however it seems to accelerate closer to the throat. This as evident from the changing slope of the black dashed profiles. It is suspected that the varying cross section of the venturi could be responsible for the observed acceleration. Across this travelling discontinuity, the velocity in the laboratory frame of reference can jump from 2.5ms$^{-1}$ to 7.5 ms$^{-1}$ over the nominal shock-front thickness of $\sim$ 2.5 mm, for the given flow conditions (see Fig. 15). The shock-front spans over 4 PIV interrogation windows. The finite thickness of the shock-front could be related to the fact that the vapour cavity is a collection of cavitation bubbles. Hence, the shock front thickness should depend on the length scale of collapsing bubbles in the vapour cavity (Brennen 1995). The velocity of the wave-front can also be approximated from the slope of the linear part of the discontinuity ($x \sim$ 12 –27 mm), in the $x-t$ diagram (see black dashed line in Fig. 14e). It is estimated to be about − 0.57$U_{t}$, which is lower than the maximum reverse flow velocity of − 0.78$U_{t}$ as shown in Fig. 15.

Further, we observe that in a few shedding cycles (see cycles marked D in Fig. 14c), although a strong re-entrant flow exists below the cavity, the rate of vaporization at such flow conditions is high. This allows the vapour cavity to continue growing, despite the presence of the re-entrant flow. Therefore, the re-entrant jet cannot dictate the cloud shedding. Instead, we can observe a typical re-entrant jet initiated shedding cycle ‘within’ a bubbly shock initiated cavity shedding cycle (see again region marked D in Fig. 14c). Further, the vapour cavity continues to grow beyond an axial position (*x*), where the adverse pressure gradient is not strong enough to drive the liquid flow upstream. Hence, a nearly stagnant pool of liquid is seen below the cavity. Ultimately, an upstream travelling wave of higher velocity ($-\overline{u_{x}} \sim 0.64U_{t}-0.75U_{t}$) arrests the growth of the vapour cavity, resulting in cavity detachment and subsequent shedding. As the focus of this study was on the re-entrant jet regime, no tomographic imaging was performed in this regime, hence we do not have an estimate for its thickness.

## Conclusions

The ‘re-entrant jet’ is known to play a key role in the cloud cavitation instability. However, the exact physical mechanism governing this phenomenon remains obscure. One of the main aims of this work was thus to delve into the re-entrant jet dynamics and assess its role in the periodic cloud shedding in an axisymmetric venturi. This is realized via multiple flow measurement modalities, i.e. high-speed shadowgraphy, tomographic imaging, and planar PIV. The shadowgraphs help us to identify the upstream travelling flow structures due to the re-entrant jet in the S-2 and S-3 stages of cavity growth in a shedding cycle. Thus, we probed into these stages to unveil the underlying re-entrant jet dynamics.

We employ tomographic imaging with the primary aim of measuring the spatio-temporal variation of the thickness and the velocity of the re-entrant liquid film in the R2 flow regime. The axisymmetry of the venturi and the re-entrant flow is exploited to gain optical access to the flow from the front side. Further, fluorescent tracer particles are used to circumvent the issue of strong reflections and opacity arising from vapour cavity and frothy mixture. Moreover, a conditional phase-averaging methodology is adopted to study the temporal evolution of re-entrant flow in a shedding cycle. The phase-averaged reconstructed particle intensity projections are used to visualise the re-entrant flow, while the standard deviation corresponding to the phase-averaged particle image intensity is used to quantify the thickness of the re-entrant jet. It appears that as the vapour cavity grows, the re-entrant flow is continuously fed below the vapour cavity, contrary to the previous understanding that it is periodically generated. Further, it is estimated that the maximum re-entrant flow thickness is about 1.2 mm for the given flow condition, i.e. 26% of the vapour cavity thickness.

The velocity vector fields reveal that the re-entrant jet is a consequence of an impinging jet and a stagnation point formed at the cavity closure region. Moreover, the velocity of the jet starting further away from the throat is higher. This suggests that the reverse flow involved in cavity detachment is driven by an adverse pressure gradient at the cavity closure region and not correlated to the pressure peaks due to the cloud collapse. The maximum velocity of the re-entrant flow below an attached cavity is found to be 0.5$U_{t}$. Further, the validity of the velocity measurements has been evaluated by checking the conservation of mass, which was deemed to be within acceptable limits. Nevertheless, the gradients close the venturi wall and air cavity cannot be resolved due to the limited particle seeding and relatively large particle size.

It is hypothesised that the thickness of re-entrant flow with respect to the vapour cavity is an important parameter, in line with Callenaere et al. (2001). At the highest cavitation number (R1), we suspect that the vapour cavities are thin enough with respect to the re-entrant flow to interact strongly with it. This prevents the re-entrant flow from reaching the throat, leading to the fragmentation of the cavity at multiple points from the cavity closure region. Hence, the upstream part of the cavity remains attached to the throat despite the continuous re-entrant flow below the cavity. As the cavitation number is decreased (R2), longer and thicker vapour cavities are formed. This limits the interaction of the re-entrant flow with the cavity. Hence, a coherent liquid flow can sustain below the vapour cavity for a large part of the shedding cycle. The re-entrant flows evolves in time, i.e. it gets thicker with respect to the vapour cavity. This allows the re-entrant flow to interact with the vapour cavity, resulting in its pinch-off at $x \sim x_{b}$. This is corroborated by an increase in the maximum re-entrant jet velocity from $0.25U_{t}$ to $0.5U_{t}$ as the vapour cavity grows in time. Thus, simultaneous measurement of the re-entrant flow thickness and the velocity reveal that a complex spatio-temporal evolution of near-wall re-entrant flow is involved in the cavity detachment. Further, the imploding cavitation cloud gives rise to a high velocity travelling wave that appears to be superimposed on the existing re-entrant flow. However, this travelling wave is deemed not necessary for the cavity detachment process in R2. Lastly, a sharp and distinct upstream travelling discontinuity is observed in the axial velocity at the lowest cavitation number (R3 regime). This is attributed to a bubbly shock wave emanating from the cloud collapse. This bubbly shockwave is superimposed on the existing pressure gradient driven re-entrant flow, creating a periodic travelling wave of a higher velocity, $0.64U_{t}-0.75U_{t}$. Moreover, this discontinuity is seen to dictate the periodic cloud detachment and shedding.

Thus, tomographic imaging followed by velocimetry of near wall ‘re-entrant flow’ has helped to further uncover the complex interaction between the near-wall flow and the vapour cavity. This deepens our understanding of the observed vapour cavity shedding behaviour, omnipresent in various industrial and maritime applications.

## Supplemental material

Movie S1 shows the phase-averaged time evolution of the vapour cavity and the re-entrant flow beneath it, in the ‘S2’ stage of the cavity growth.

### Supplementary Information

Below is the link to the electronic supplementary material.

Supplementary file1 (AVI 67,650kb)

## Figures and Tables

**Fig. 16:**
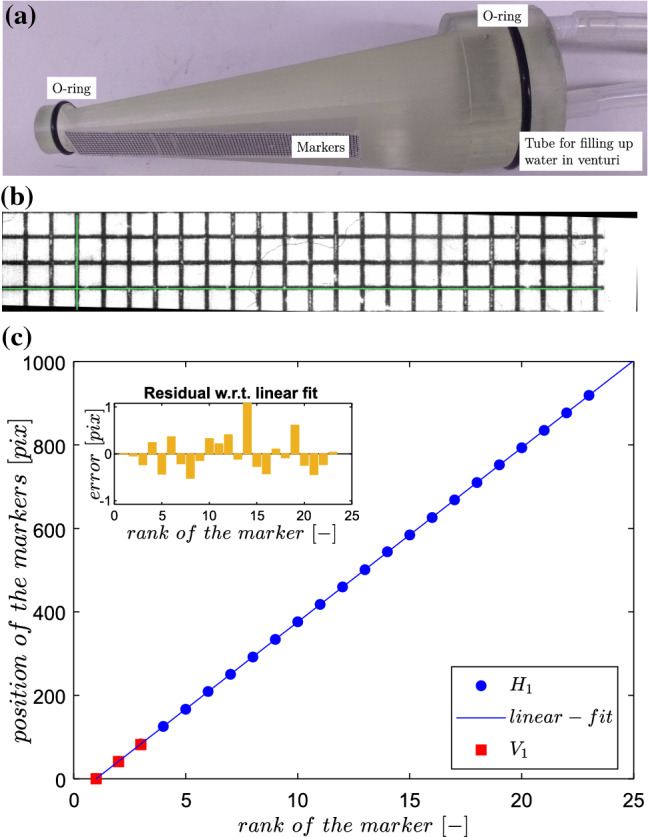
**a** 3-D printed venturi negative with calibration target. **b** A sample calibration image of marker (‘+’). **c** Positions of markers detected by a 3 point Gaussian fit to the intensity in horizontal direction ($H_{1}$) and vertical direction ($V_{1}$). The least-square linear fit is applied to $H_{1}$ data points and residual errors are shown in the inset

**Fig. 17:**
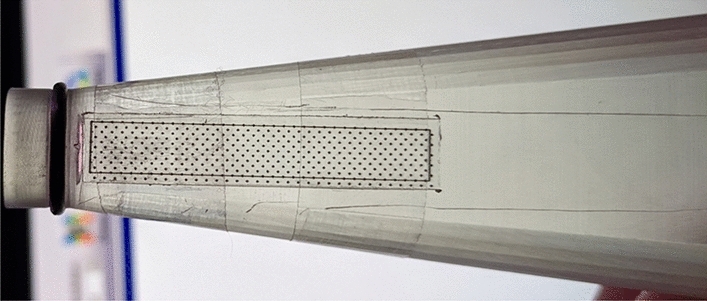
3-D printed venturi negative with calibration target for tomographic imaging

## Appendix: Calibration

The calibration for planar PIV measurement is performed using a calibration target attached to a 3-D printed venturi negative. The markers (‘+’) with known spacing are shown in Fig. 16a. The markers are closely spaced (i.e. next to each other). Hence, it appears as a grid. The target is carefully slid inside the venturi, which is held in place by a set of O-rings on either ends. This part of the flow loop, with venturi, is then filled with water. A sample calibration image is shown in Fig. 16b. The LED panel is used to illuminate the markers from the backside.

The positions of detected markers (in pixels) are plotted in horizontal and vertical direction, shown by blue and red symbols, respectively (see Fig. 16c). It is seen that the magnification in vertical and horizontal directions is identical. Moreover, there is no image warping as evident by a linear fit to the detected markers, where the residual error is within 1.5 pixels (illustrated in the inset of Fig. 16c). This is expected as the camera plane is parallel to the calibration target (see experimental setup in Figs. 4 and 5).

A similar approach is employed for the calibration of tomographic imaging. Due to the restricted access to the FOV, traversing of the calibration target inside the venturi in the *z*-direction was impractical. Hence, 3-D calibration was performed using two available *z*-planes. The calibration markers (‘$\bullet$’) were laser-printed on each side of a glass slide to provide two calibration planes. Further, the markers were staggered to identify the appropriate plane in the calibration image (see Fig. 17). This calibration target is then attached on the 3-D printed venturi negative such that the markers can be placed at the desired location of measurement. The calibration is further improved by using self-calibration, as discussed in the manuscript.

## Footnotes

1 in terms of cost, opacity, ‘non-invasiveness’

## References

[CR1] Barbaca L, Pearce BW, Ganesh H, Ceccio SL (2019). On the unsteady behaviour of cavity flow over a two-dimensional wall-mounted fence. J Fluid Mech.

[CR2] Barre S, Rolland J, Boitel G, Goncalves E, Fortes Patella R (2009). Experiments and modeling of cavitating flows in venturi: attached sheet cavitation. Eur J Mech B/Fluids.

[CR3] Brennen Christopher E (1995). Cavitation and bubble dynamics.

[CR4] Brunhart M, Soteriou C, Gavaises M, Karathanassis I, Koukouvinis P, Jahangir S, Poelma C (2020). Investigation of cavitation and vapor shedding mechanisms in a Venturi nozzle Maxwell. Phys Fluids.

[CR5] Budich B, Schmidt SJ, Adams NA (2018). Numerical simulation and analysis of condensation shocks in cavitating flow. J Fluid Mech.

[CR6] Callenaere M, Franc JP, Michel JM, Riondet M (2001). The cavitation instability induced by the development of a re-entrant jet. J Fluid Mech.

[CR7] Coutier-Delgosha O, Stutz B, Vabre A, Legoupil S (2007). Analysis of cavitating flow structure by experimental and numerical investigations. J Fluid Mech.

[CR8] De Lange DF, De Bruin GJ (1997). Sheet cavitation and cloud cavitation, re-entrant jet and three-dimensionality. Flow Turbul Combust.

[CR9] Dular M, Bachert R, Stoffel B, Širok B (2005). Experimental evaluation of numerical simulation of cavitating flow around hydrofoil. Eur J Mech B/Fluids.

[CR10] Elsinga GE, Scarano F, Wieneke B, Van Oudheusden BW (2006). Tomographic particle image velocimetry. Exp Fluids.

[CR11] Fitzgerald Janice A, Garimella Suresh V (1997) Visualization of the flow field in a confined and submerged impinging jet. American Society of Mechanical Engineers, Heat Transfer Division, (Publication) HTD, 346:93–96. ISSN 02725673

[CR12] Foeth EJ, Van Doorne CWH, Van Terwisga T, Wieneke B (2006). Time resolved PIV and flow visualization of 3D sheet cavitation. Exp Fluids.

[CR13] Franc JP (2001) Partial Cavity Instabilities and Re-Entrant Jet. International Symposium on Cavitation, (Tsujimoto)

[CR14] Furness H (1975) Experimental and Theoretical Studies of Two-Dimensional Fixed-Type Cavities. Journal of Fluids Engineering, Transactions of the ASME

[CR15] Ganesh H, Simo MA, Steven LC (2016). Bubbly shock propagation as a mechanism for sheet-to-cloud transition of partial cavities. J Fluid Mech.

[CR16] Giannadakis E, Gavaises M, Arcoumanis C (2008). Modelling of cavitation in diesel injector nozzles. J Fluid Mech.

[CR17] Gnanaskandan A, Mahesh K (2016). Large Eddy simulation of the transition from sheet to cloud cavitation over a wedge. Int J Multiph Flow.

[CR18] Gopalan S, Katz J (2000). Flow structure and modeling issues in the closure region of attached cavitation. Phys Fluids.

[CR19] Jahangir S, Hogendoorn W, Poelma C (2018). Dynamics of partial cavitation in an axisymmetric converging-diverging nozzle. Int J Multiph Flow.

[CR20] Jahangir S, Wagner EC, Mudde RF, Christian P (2019). Void fraction measurements in partial cavitation regimes by X-ray computed tomography. Int J Multiph Flow.

[CR21] Jyoti KK, Pandit AB (2004). Ozone and cavitation for water disinfection. Biochem Eng J.

[CR22] Kawanami Y, Kato H, Yamaguchi H, Tanimura M, Tagaya Y (1997). Mechanism and control of cloud cavitation. J Fluids Eng Trans ASME.

[CR23] Kim H, Westerweel J, Elsinga GE (2013). Comparison of Tomo-PIV and 3D-PTV for microfluidic flows. Meas Sci Technol.

[CR24] Knapp RT (1958). Recent investigations of the mechanics of cavitation and cavitation damage. Wear.

[CR25] Kubota A, Kato H, Yamaguchi H, Architecture Naval (1989) Unsteady Structure Measurement of Cloud Cavitation on a Foil Section Using Conditional Sampling Technique. Journal of Fluids Engineering, Transactions of the ASME, 111

[CR26] Kuiper G (1997). Cavitation research and ship propeller design. Appl Sci Res (The Hague).

[CR27] Laberteaux K, Ceccio S (2001). Partial cavity flows. Part 1. Cavities forming on models without spanwise variation. J Fluid Mech.

[CR28] Laberteaux KR, Ceccio SL (2001). Partial cavity flows. Part 2. Cavities forming on test objects with spanwise variation. J Fluid Mech.

[CR29] Le Q (1993). Partial cavities: global behavior and mean pressure distribution. J Fluids Eng Trans ASME.

[CR30] Leroux JB, Astolfi JA, Billard JY (2004). An experimental study of unsteady partial cavitation. J Fluids Eng Trans ASME.

[CR31] Lush PA, Skipp SR (1986). High speed cine observations of cavitating flow in a duct. Int J Heat Fluid Flow.

[CR32] Nakashima K, Ebi Y, Shibasaki-kitakawa N, Soyama H, Yonemoto T (2016). Hydrodynamic cavitation reactor for efficient pretreatment of lignocellulosic biomass. Ind Eng Chem Res.

[CR33] Pelz PF, Keil T, Gro TF (2017). The transition from sheet to cloud cavitation. J Fluid Mech.

[CR34] Pham TM, Larrarte F, Fruman DH (1999). Investigation of unsteady sheet cavitation and cloud cavitation mechanisms. J Fluids Eng Trans ASME.

[CR35] Poelma C (2020). Measurement in opaque flows: a review of measurement techniques for dispersed multiphase flows. Acta Mech.

[CR36] Reuther N, Kähler CJ (2018). Evaluation of large-scale turbulent/non-turbulent interface detection methods for wall-bounded flows. Exp Fluids.

[CR37] Saito Y, Takami R, Nakamori I (2007). Numerical analysis of unsteady behavior of cloud cavitation around a NACA0015 foil. Comput Mech.

[CR38] Sakoda M, Yakushiji R, Maeda M, Yamaguchi H (2001) Mechanism of cloud cavitation generation on a 2-D hydrofoil. Fourth International Symposium on Cavitation, number 1996:1–8

[CR39] Stanley C, Barber T, Rosengarten G (2014). Re-entrant jet mechanism for periodic cavitation shedding in a cylindrical orifice. Int J Heat Fluid Flow.

[CR40] Stutz B, Reboud JL (1997). Two-phase flow structure of sheet cavitation. Phys Fluids.

[CR41] Trummler T, Schmidt SJ, Adams NA (2020). Investigation of condensation shocks and re-entrant jet dynamics in a cavitating nozzle flow by Large-Eddy Simulation. Int J Multiph Flow.

[CR42] Westerweel J (1997). Fundamentals of digital particle image velocimetry. Meas Sci Technol.

[CR43] Westerweel J, Scarano F (2005). Universal outlier detection for PIV data. Exp Fluids.

[CR44] Wieneke B (2008). Volume self-calibration for 3D particle image velocimetry. Exp Fluids.

